# Editorial: TGF-β in Human Disease: Friend or Foe?

**DOI:** 10.3389/fcell.2021.739172

**Published:** 2021-09-29

**Authors:** Guoping Zheng, David C. H. Harris

**Affiliations:** ^1^Westmead Institute for Medical Research, Sydney, NSW, Australia; ^2^Faculty of Medicine and Health, University of Sydney, Sydney, NSW, Australia; ^3^Westmead Hospital, Westmead, NSW, Australia

**Keywords:** TGF-β, inflammation, fibrosis, cancer, TCF, Foxo (forkhead box protein O), p53, SMAD

TGF-β is a family of essential multifunctional cytokines regulating (1) cell growth & development at both embryonic and adult stages; (2) inflammation and maintenance of host resistance mechanisms; and (3) remodeling and repair processes including angiogenesis and regeneration. However, conflicting roles of TGF-β in human diseases create a major challenge to the therapeutic targeting of this multifunctional cytokine.

In this issue, the protean roles of TGF-β in humans are discussed in chronic inflammatory diseases characterized with inflammation and fibrosis, in cerebrovascular disease and in cancer. The chronic inflammatory diseases included are chronic kidney disease, diabetic kidney disease, cardiac fibrosis, pulmonary arterial hypertension, atherosclerosis, and fibrosis of other organs. TGF-β-induced fibrosis of the diseased organs is one of the most prominent features of these diseases. TGF-β contributes to organ fibrosis by induction of mesenchymal transition, namely epithelial-mesenchymal transition (EMT) and endothelial-mesenchymal transition (EndoMT), although their direct contribution to the origin of myofibroblasts in organ fibrosis has been debated (Ma et al.). The potential of TGF-β-induced EndoMT in tissue engineering is reviewed. In addition, TGF-β's induction of mesenchymal transition is discussed with regard to its contribution to development of cerebrovascular diseases such as cerebral cavernous malformation due to maladaptive dysfunction of TGF-β (Zhang and Yang). Post-translational modification of SMAD ubiquitin regulatory factor 2 (Smurf2) is reviewed for its role in regulating TGF-β signaling and its potential for targeting TGF-β signaling in human fibrotic diseases and cancer (Bai and Ying). p53, a tumor suppressor, was found to complex with SMADs to transcriptionally regulate genomic TGF-β1 fibrotic-response gene profiles. SMADs/p53 targeted genes and cross-talking pathways are discussed as targets for treating kidney fibrosis (Higgins et al.). The protective anti-inflammatory roles of TGF-β via differential functions of the SMAD family members are reviewed in kidney disease (Gu et al.). New candidate targets for treatment of kidney fibrosis, receptor interacting protein kinase 3 (RIPK3) (Shi et al.) and calcium-activated potassium channel KCa3.1 (Huang et al.) are discussed. An important review article discusses in depth the lack of success of on-target anti-TGF-β therapies in clinical cancer treatment as well as remaining challenges (Teixeira et al.).

The conflicting roles of TGF-β, which may slow or accelerate progression of various human diseases, render it unsuitable as a therapeutic target. Indeed, anti-TGF-β therapies have proven unsuccessful in clinical trials for fibro-inflammatory kidney disease (Vincenti et al., 2017), and in cancer clinical trials (Ahmadi et al., 2019).

To resolve the conflicting anti-inflammatory and profibrotic roles of TGF-β in inflammatory diseases, rebalancing of Smad3/Smad7 signaling or specifically targeting Smad3-dependent non-coding RNAs that regulate kidney fibrosis or inflammation suggested as better therapeutic approaches for kidney fibrosis (Gu et al.).

Similarly in cancer, TGF-β inhibits proliferation and induces apoptosis of cancer cells, but it also promotes metastasis by inducing the invasive phenotype of tumor cells through induction of epithelial-mesenchymal transition (EMT). Furthermore, it causes immuno-suppression, including the induction of immunosuppressive checkpoint molecule PD-1, which inactivates the anti-tumor function of immune cells. Combination of anti-TGF-β therapies with immune checkpoint inhibitors, or more spatio-temporal controlled interventions, are suggested by Teixeira *et al* for improved treatment of cancer (Teixeira et al.).

Previously, a mechanism to explain the multiple functions of TGF-β had been proposed by Massagué. He suggested that the net outcome of TGF-β signals is determined by a cell specific complex network of cross-talking signals. The combined DNA-binding specificity of a SMAD-cofactor complex dictates the choice of target gene, whereas SMAD affinity for DNA is too low to do that alone (Massague, 2000). β-catenin binding to SMAD as a cofactor enables a cell specific cross-talk between TGF-β and Wnt pathways. Foxo has been identified as another co-factor of β-catenin competing with TCF.

β-catenin binds to either TCF or Foxo to produce opposite signaling outcomes: proliferation (β-catenin/TCF) or cell cycle arrest for survival under oxidative stress (β-catenin/Foxo). TGF-β mediates pro-fibrotic signaling via cross-talk with multiple pathways including Wnt/β-catenin, integrin/integrin linked kinase (ILK), the renin angiotensin system (RAS), Notch/NICD and mROR, all converging at activation of β-catenin/TCF (Figure 1). In this context, Qiao and colleagues revealed in murine models of kidney fibrosis that the inhibition of β-catenin/TCF prevented the profibrotic effects of TGF-β while promoting its anti-inflammatory function via concomitant β-catenin/Foxo1-induction of regulatory T cells (Tregs) (Qiao et al., 2018, Rao et al., 2019, Rao et al., 2021; Figure 1). This has been confirmed by other labs; the β-catenin/Foxo complex induces Tregs (Sumida et al., 2018) and protects against kidney fibrosis (Nlandu-Khodo et al., 2020).

**Figure 1:**
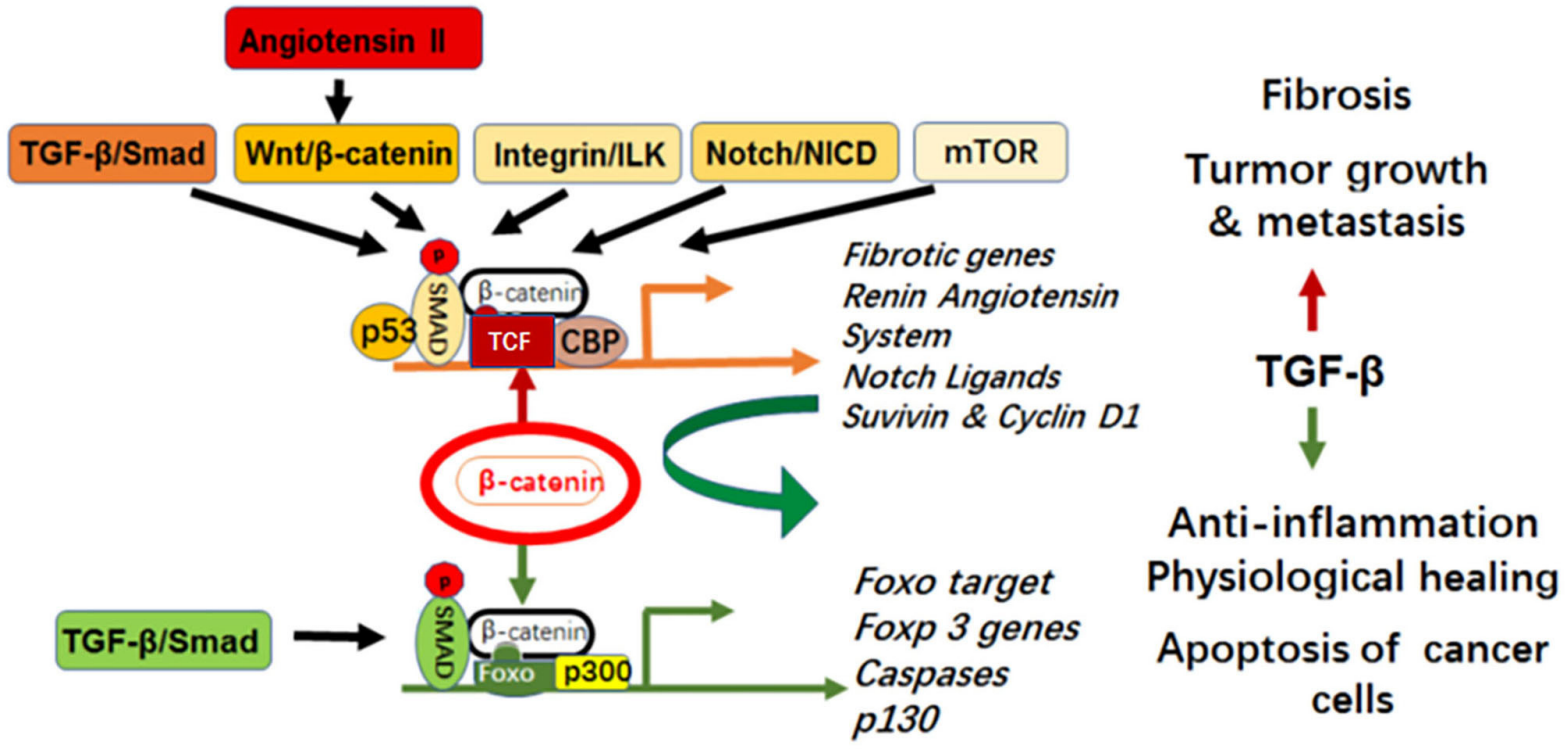
Cell specific cross-talking pathways in fibro-inflammatory disease and cancer.

In summary, the papers in this issue illustrate how the various paradoxical functions of TGF-β signaling may be targeted to design and optimize therapeutic approaches for patients suffering from diseases associated with TGF-β. More importantly, dissection of the unique and often conflicting roles of transcription cofactor complexes holds great promise for targeting TGF-β pathways in human disease (Emami et al., 2004).

## Author Contributions

GZ contributed to conception and drafting and final approval of the manuscript. DH contributed to conception and final approval of the manuscript. All authors contributed equally to the article and approved the submitted version.

## Funding

GZ and DH are supported by NHMRC project grant 1141235 and investigator grant 1195473.

## Conflict of Interest

The authors declare that the research was conducted in the absence of any commercial or financial relationships that could be construed as a potential conflict of interest.

## Publisher's Note

All claims expressed in this article are solely those of the authors and do not necessarily represent those of their affiliated organizations, or those of the publisher, the editors and the reviewers. Any product that may be evaluated in this article, or claim that may be made by its manufacturer, is not guaranteed or endorsed by the publisher.
